# Establishing the acute physiological and sleep disruption characteristics of wind farm versus road traffic noise disturbances in sleep: a randomized controlled trial protocol

**DOI:** 10.1093/sleepadvances/zpad033

**Published:** 2023-09-06

**Authors:** Gorica Micic, Branko Zajamsek, Bastien Lechat, Kristy Hansen, Hannah Scott, Barbara Toson, Tessa Liebich, Claire Dunbar, Duc Phuc Nguyen, Felix Decup, Andrew Vakulin, Nicole Lovato, Leon Lack, Colin Hansen, Dorothy Bruck, Ching Li Chai-Coetzer, Jeremy Mercer, Con Doolan, Peter Catcheside

**Affiliations:** Flinders University, Flinders Health and Medical Research Institute: Sleep Health, College of Medicine and Public Health, Australia; Flinders University, Flinders Health and Medical Research Institute: Sleep Health, College of Medicine and Public Health, Australia; Flinders University, Flinders Health and Medical Research Institute: Sleep Health, College of Medicine and Public Health, Australia; Flinders University, College of Science and Engineering, Australia; Flinders University, Flinders Health and Medical Research Institute: Sleep Health, College of Medicine and Public Health, Australia; Flinders University, Flinders Health and Medical Research Institute: Sleep Health, College of Medicine and Public Health, Australia; Flinders University, College of Education, Psychology and Social Work, Australia; Flinders University, College of Education, Psychology and Social Work, Australia; Flinders University, College of Science and Engineering, Australia; Flinders University, College of Science and Engineering, Australia; Flinders University, Flinders Health and Medical Research Institute: Sleep Health, College of Medicine and Public Health, Australia; University of Sydney, NEUROSLEEP, Woolcock Institute of Medical Research, Australia; Flinders University, Flinders Health and Medical Research Institute: Sleep Health, College of Medicine and Public Health, Australia; Flinders University, Flinders Health and Medical Research Institute: Sleep Health, College of Medicine and Public Health, Australia; Flinders University, College of Education, Psychology and Social Work, Australia; The University of Adelaide, School of Mechanical Engineering, Australia; Victoria University, Institute for Health and Sport, Australia; Flinders University, Flinders Health and Medical Research Institute: Sleep Health, College of Medicine and Public Health, Australia; Department of Respiratory, Sleep Medicine and Ventilation, Southern Adelaide Local Health Network, SA Health, Australia; Department of Respiratory, Sleep Medicine and Ventilation, Southern Adelaide Local Health Network, SA Health, Australia; University of New South Wales, School of Mechanical and Manufacturing Engineering, Australia; Flinders University, Flinders Health and Medical Research Institute: Sleep Health, College of Medicine and Public Health, Australia

**Keywords:** wind farm, wind turbine, environment noise, road traffic, sleep quality, sleep disturbance, sleep disruption, electroencephalogram

## Abstract

**Study Objectives:**

Despite the global expansion of wind farms, effects of wind farm noise (WFN) on sleep remain poorly understood. This protocol details a randomized controlled trial designed to compare the sleep disruption characteristics of WFN versus road traffic noise (RTN).

**Methods:**

This study was a prospective, seven night within-subjects randomized controlled in-laboratory polysomnography-based trial. Four groups of adults were recruited from; <10 km away from a wind farm, including those with, and another group without, noise-related complaints; an urban RTN exposed group; and a group from a quiet rural area. Following an acclimation night, participants were exposed, in random order, to two separate nights with 20-s or 3-min duration WFN and RTN noise samples reproduced at multiple sound pressure levels during established sleep. Four other nights tested for continuous WFN exposure during wake and/or sleep on sleep outcomes.

**Results:**

The primary analyses will assess changes in electroencephalography (EEG) assessed as micro-arousals (EEG shifts to faster frequencies lasting 3–15 s) and awakenings (>15 s events) from sleep by each noise type with acute (20-s) and more sustained (3-min) noise exposures. Secondary analyses will compare dose–response effects of sound pressure level and noise type on EEG K-complex probabilities and quantitative EEG measures, and cardiovascular activation responses. Group effects, self-reported noise sensitivity, and wake versus sleep noise exposure effects will also be examined.

**Conclusions:**

This study will help to clarify if wind farm noise has different sleep disruption characteristics compared to road traffic noise.

**Statement of Significance**

Wind farms are expanding globally, but ongoing public concern and uncertainty remains regarding potential impacts on human sleep and health. Laboratory studies using objective measures of sleep and comparisons of wind farm versus road traffic noise, known to disrupt sleep, are lacking and warranted. This project sought to use direct physiological measurements to quantify the impact of wind turbine noise compared to road traffic noise on sleep. Individuals living near and far from environmental noise sources were studied in carefully controlled laboratory conditions using prerecorded wind farm noise. Study findings will help to inform if wind farm noise has different effects on sleep compared to road traffic noise to support evidence-based noise level guidelines for healthy sleep in residents living near wind farms.

## Introduction

Wind farms continue to gain prominence as a major source of sustainable energy generation in over 90 countries [1]. However, onshore wind farms are also a source of public concern and debate regarding potential adverse effects of wind farm noise (WFN) emissions on nearby communities. Sufficiently loud noise from any source has the potential to disturb sleep, health and well-being through direct sleep disruption effects, and through inherent or acquired noise sensitivity and annoyance effects that could promote insomnia [2]. Predominantly through their large size and complex aero-acoustic effects including blade passage past the turbine tower, wind turbine noise emissions are dominated by low frequencies including infrasound [3], which is defined as low frequency noise < 20 Hz; below the conventionally accepted lower frequency limit of average human hearing from 20 Hz and 20 kHz [4]. Although individuals with above average low frequency hearing acuity can potentially hear WFN infrasound within a few hundred meters of a wind turbine, WFN infrasound is unlikely to be audible at longer-range distances. Given the importance of hearing for sensing sound, prominent audible WFN features appear much more likely to negatively impact on sleep compared to inaudible infrasound [5].

The World Health Organization (WHO) concluded that there is overwhelming evidence that exposure to environmental noise has adverse effects on population health. Per annum in Europe, noise pollution is estimated to contribute 1.0–1.6 million healthy life years lost, including 900 000 healthy life years lost through sleep disturbance [6]. Accordingly, to help protect sleep, WHO environmental noise guidelines [6] and allowable noise limits across many jurisdictions [7] suggest that outdoor sound pressure levels (SPLs) at night produced by traffic and other sources, including wind farms, should not exceed an A-weighted equivalent level (L_Aeq_) of 40 dB. Provided the building structure is substantial and windows are closed, outside noise can be attenuated by around 10–15 dB, resulting in indoor noise levels of around 30 dB(A) [6, 8]. These recommendations reflect evidence accumulated primarily from road traffic, railway and aircraft effects based on noise level measurements A-weighted to average human hearing curves on the logarithmic dB scale. However, WFN has substantially different acoustic characteristics compared to road, rail, and air traffic noise, including much more predominant low frequency and time-varying noise features which could have different impacts on sleep. Thus, A-weighted noise criteria derived from traffic noise may not be entirely appropriate for WFN, particularly given substantial inter-individual variability in low frequency hearing acuity and more compressed equal-loudness contours at lower frequencies that could potentially render low frequency noise inaudible to some and yet clearly audible and sleep disruptive to others.

Modern WFN is dominated by low frequencies (<200 Hz) at noise propagation distances beyond a few hundred meters most relevant to typical human exposure in neighboring households. Road, rail, and air traffic noise also contain some low frequencies, but are predominantly mid-high frequency (>200 Hz) noise that is substantially more attenuated over distance and by intervening objects compared to low frequency dominated noise. WFN, and particularly prominent aero-acoustic effects from blade-rotation, including dynamically changing lift and potentially stall, contribute to time-varying amplitude modulation (AM). Tonal AM, most likely mechanical in origin, has also been observed in the context of wind farm operation, and the associated low-frequency components have been measured at audible SPLs up to several kilometers from the source [9, 10]. Noise with AM is consistently rated as more annoying compared to noises of equivalent A-weighted SPL without AM [11]. At close distances (< 2 km from the nearest wind turbine), WFN with AM is often described as “swish,” but at greater distances, “rumbling” or “thumping” [12] is a more common description.

WFN characteristics are influenced by many factors such as the number, type and size of turbines; distance from the source; background noise levels which are typically low in rural areas (particularly at night); local topology; wind speed and direction; atmospheric temperature profile (including inversions); turbulence conditions; and the nature and characteristics of intervening structures that impact audibility and perception. Consequently, WFN is highly variable and can be sporadic or persistent, which potentially makes habituation to WFN more difficult compared to more consistent and predictable (e.g. heavy road traffic) or transient (e.g. more sporadic traffic pass-bys) noise sources. Furthermore, unlike traffic noise, which is typically reduced at night when traffic volumes are typically lower, more stable environmental conditions are often favorable to ongoing WFN propagation at night when other background noise and wind-speeds in neighboring lower lying residential locations are usually lowest. Thus, when present, prominent WFN at night has significant potential to disturb sleep of neighboring residents.

Excessive noise from neighborhood, traffic and industrial noise sources is one of the most common public complaints, and an established cause of annoyance, stress, raised blood pressure, sleep disturbance, related health impacts and pharmaceutical use [6, 13, 14]. In terms of sleep macrostructure, nocturnal noise causes sleep fragmentation, resulting in shallower sleep from increased arousals and redistribution of sleep architecture (i.e. increased light sleep (wake and stage 1 sleep) and decreased slow wave sleep and REM sleep [15–18]. Auditory arousal thresholds measured during N2, deep and REM sleep, in good sleepers and those with sleep onset insomnia do not appear to systematically differ, despite insomniacs reporting being “light sleepers” [19]. This suggests that noise-related sleep disturbance may not be substantially different between individuals, even in those with insomnia [20]. Nevertheless, as has previously been supported by several studies [21–23], a vulnerability to stress-induced sleep disturbance clearly has the potential to lead to transient and potentially more chronic conditioned insomnia.

On a microstructural level, cortical electroencephalographic (EEG) response probability and magnitude also depend largely on the type and intensity of a noise stimulus and on sleep depth [24–27]. In addition to cortical EEG responses, traffic noise is known to trigger autonomic or “sub-cortical” reflex responses in sleep [26]. These reflexes rapidly augment cardiovascular, respiratory and metabolic activity in preparation for behavioral “flight or fight” responses. This includes a blood pressure surge through increased heart rate, and a particularly prominent skin vasoconstriction response readily discernible as attenuation in finger pulse oximeter waveform amplitude [25]. Sensory disturbances producing no visually discernible EEG changes can still produce a clear reflex cardiovascular response. There is also some evidence to support that frequent noise-induced cardiovascular responses without more frequent EEG arousals negatively impacts next-day sleepiness and mood [28]. Thus, it is important to consider that noise-induced sleep disturbances may have important effects on daytime functions.

Although extensive literature regarding environmental noise emitted from road, rail and air exists, important knowledge gaps remain regarding wind farm noise effects on sleep [2]. Data from well-designed studies using objective measures of sleep under carefully controlled noise conditions are particularly scarce. In some of the most comprehensive studies to date, Persson Waye et al. found that amplitude-modulated continuous WFN exposure produced small but statistically significant reductions in self-assessed sleep quality and some aspects of EEG evaluated sleep [29–31]. Using sleep actigraphy data from around 250 individuals studied over multiple nights, Michaud et al also found some evidence to support small increases in overnight movement time in response to changes in wind farm sound pressure levels [32]. Thus, transient noise events and time-varying features of WFN may be more sleep disruptive than continuous noise.

Adverse effects of WFN exposure have also been attributed to infrasound, but without supporting evidence. A previous study found no discernible EEG changes with overnight exposure to 10 Hz infrasound at 105 dB [33]. A more recent study, using 72 h of simulated wind farm infrasound exposure, also found no evidence to support any discernible effects on sleep [34]. However, no previous study has directly evaluated if wind farm noise, including infrasound, and prominent audible amplitude modulated components, is potentially more sleep disruptive compared to road traffic noise when replayed under carefully controlled laboratory conditions needed to avoid a wide-range of potential confounders.

This paper outlines a study protocol designed to compare the sleep disruption effects of wind farm versus road traffic noise on established sleep, and to examine the impact of wind farm noise exposure during wake and/or sleep on conventional overnight measures of sleep time and quality in individuals with and without prior wind farm noise exposure and noise-related sleep complaints.

The effects of environmental noise on sleep are best tested using direct EEG measures of sleep on cortical activity. During sleep, cortical responses to sensory stimuli are markedly diminished. However, brainstem mechanisms continue to process sensory stimuli, with thalamic “gating” of physiological responses according to stimulus salience and intensity and the depth of sleep itself. Thus, the effects of auditory stimuli on sleep depend on the type and intensity of the noise stimulus and on the depth of sleep during which a noise stimulus occurs. Responses to noise can range from no discernible response in the EEG or any other physiological signal through to full awakening (shifts to faster EEG frequencies > 15 s), but can also include increased micro-arousals (3–15 s shifts toward faster EEG frequencies), reflex cardiovascular responses, and K-complexes in the EEG. On the sleep macrostructure level, preexisting stress and extraneous noises can impair sleep initiation and the return to sleep after waking to reduce total sleep time and sleep efficiency (the percentage of the sleep opportunity occupied by sleep). Thus, carefully controlled laboratory studies of EEG and cardiovascular activation responses to noise exposure during sleep allow for robust evaluation of WFN specific effects on sleep with a reduced risk of confounding from a range of potential biases in real-world noise exposure settings.

### Aims and hypotheses

This study sought to clarify the effects of WFN on sleep compared to RTN, an already known disruptor to sleep, and quiet background noise (control). The primary study aims were to compare the dose–response effects of WFN versus RTN on:

1. The probability of EEG-defined micro-arousals and awakenings from sleep (shifts to faster EEG frequencies for ≥ 3 and ≥ 15 s, respectively) under each noise condition on a 20-second noise battery night to assess the acute noise effects.
2. The probability of EEG-defined micro-arousals and awakenings from sleep under each noise condition on a 3-minute noise battery night to assess more sustained noise effects.

The hypotheses for the two primary aims were that:

1. EEG arousal responses are more probable with brief 20-second WFN compared to RTN exposures of equivalent A-weighted SPL.
2. EEG arousals responses, including longer periods of wake, are more probable with more prolonged 3-minute WFN compared to RTN exposures of equivalent A-weighted SPL.

The study was also designed to address the following secondary aims to:

1. Examine the role of wake-related noise exposure prior to sleep onset on objective and subjective measures of sleep disruption and next-day mood, anxiety, sleepiness, and daytime performance, by presenting WFN noise only during wake, only during sleep, and continuously during both wake and sleep throughout separate overnight sleep opportunities.
2. Examine the role of habitual noise exposure history and self-reported noise sensitivity on objective and subjective sleep in four pre-existing populations: individuals living near wind turbines including a group with and a group without noise-related complaints, individuals living in urban areas near road traffic, and individuals living in a quiet rural area.
3. Compare the dose–response effects of sound pressure level and noise type on the probability of EEG (K-complexes and quantitative electroencephalography measures) and cardiovascular activation responses (tachy-brady cardias, finger vasoconstriction and pulse arrival time) using established methods [35].
4. Examine the dose–response effects of sound pressure level and noise type on daytime listening test outcomes of self-reported annoyance and perceived acceptability for sleep.

## Methods

### Study design

**Figure 1:**
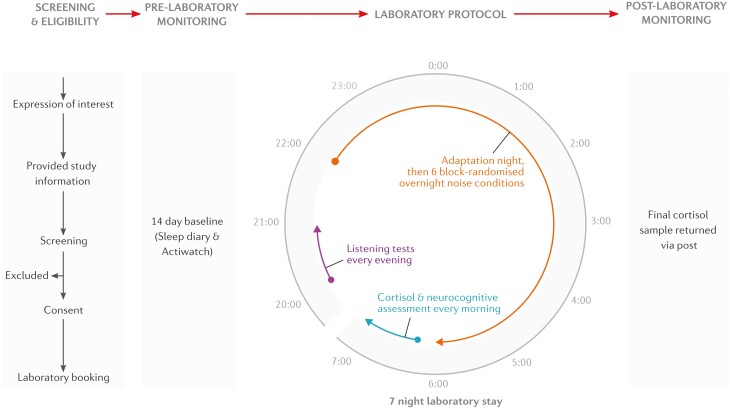
Flow chart of participant involvement from initial contact to end of testing procedures.

This manuscript describes the pre-planned protocol for a recently-completed randomized, cross-over, mixed-subjects (noise exposure condition: within-subjects, participant group: between-subjects) interventional trial. Four participant groups determined by residential proximity to noise sources and self-reported noise-related complaints were exposed to six randomized, counter-balanced overnight noise exposure conditions after an adaptation night (seven nights total). The four participant groups were 1) wind turbine noise exposed with noise-related complaints (WFN1), 2) wind turbine noise exposed without noise-related complaints (WFN2), 3) road traffic noise exposed with noise-related complaints (RTN), and 4) rural area controls (CN). The six noise exposure conditions were WFN only when in established (N2, N3, REM) sleep (1), WFN only when awake or transitional N1 sleep (2), WFN throughout the sleep opportunity (3), 20-second WFN and RTN noise samples only when in established sleep (4), 3-minute WFN and RTN noise samples only when in established sleep (5), and a quiet control night (6). Figure 1 summarizes the study data collection processes.

### Facilities

Clinical audiology assessments were conducted at the Flinders University Hearing Services (Department of Speech Pathology and Audiology, Flinders Medical Centre, Bedford Park, South Australia). Experimental aspects were conducted at the Nick Antic Sleep Laboratory (Adelaide Institute for Sleep Health, Mark Oliphant Building, Bedford Park, South Australia). This six-bedroom facility has two bedrooms purpose built for low frequency noise and infrasound reproduction, achieving very low night-time background noise levels of approximately 19 dBA. These are fully contained, private, sound attenuated bedrooms with a king-single bed, individual en-suites and a shared participant lounge area. The bedrooms were temperature controlled by room thermostats set to 23°C. Lights were dimmed (to < 10 lx) in the evening prior to bedtime, turned off between lights-out and lights-on time (<1 lx), and at normal levels (~150 lx) according to wake demands at all other times.

### Participants

Ethical approval was obtained from the Southern Adelaide Clinical Human Research Ethics Committee (project number 343.18) and governance approval from the local research governance office at the Flinders Medical Centre.

Eligible individuals were recruited via advertisements posted on websites, media outlets, and public noticeboards, via community engagement talks and word of mouth, and from a sample of participants who undertook a computer assisted telephone interview (CATI) survey as part of a related project.

#### Inclusion criteria

- Aged ≥ 18 years.
- Able to understand study commitments and provide informed consent in writing.
- Able and willing to travel and remain at the laboratory for seven nights.

One of the following (to determine participant group allocation):

◦ Living < 10 km from a wind turbine and self-reporting WFN-related sleep disturbance.
◦ Living < 10 km from a wind turbine and self-reporting no WFN-related sleep disturbance.
◦ Living close to a main road and self-reporting RTN-related sleep disturbance.
◦ Living in a rural area.

#### Exclusion criteria

- Language difficulties precluding fully informed consent.
- A known diagnosis of a sleep disorder other than Insomnia (e.g. Obstructive Sleep Apnoea, Restless Legs Syndrome) based on self-reported screening questions. Insomnia was not excluded as this would risk substantial bias against the potentially most relevant individuals with noise-related sleep difficulties.
- Pregnancy/lactation.
- Night shift work (work hours between 10 pm and 6 am) or trans-meridian travel (equal to or greater than two time zones) in the last 2 months.
- Significant hearing difficulties (≤60 dB), based on self-reported screening questions then confirmed during an extensive hearing threshold assessment by an independent audiologist (pure tone audiometry between 125 and 8000 Hz in each ear in a hearing booth).

### Participant enrolment and consent

After completing an online screening questionnaire, participants who meet inclusion/exclusion criteria were invited to participate in the main laboratory trial. Once study eligibility was established, study personnel obtained informed consent from each participant and scheduled the seven-night laboratory stay, subject to participant, facility, and experimenter availability.

### Interventions

**Figure 2:**
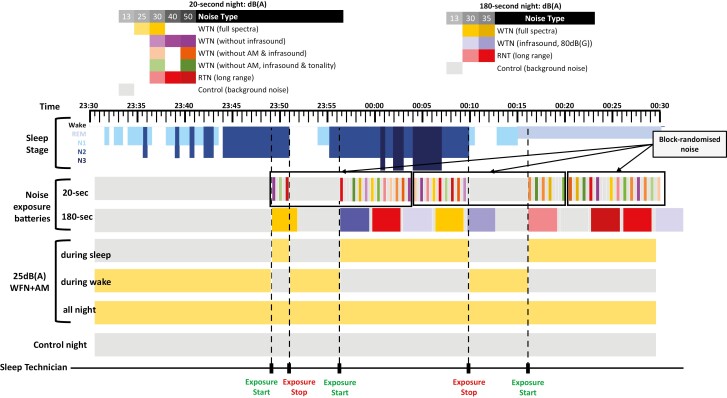
One-hour “snapshot” schematic representation of the 6 randomized laboratory intervention nights (not including the first adaptation night). Time (i.e., in 30-second epochs) is represented across the top and simulated sleep stages (wake, N1, 2, 3 and REM) measured by polysomnography are shaded in blue. 20-second and 3-minute noise batteries were played in block-randomized order after 5-min of N2/REM sleep and 1-minute of re-established N2/REM sleep thereafter. During the three intervention nights playing WFN at typical indoor levels, experimental exposure will cease immediately at wake/N1 sleep, besides the fixed prolonged noise battery exposure in which noise will play for the full 3-min or 20-s.

All participants completed an adaptation night (no noise) on the first evening of their laboratory stay followed by six overnight noise exposure conditions in block-randomized order (Figure 2). The six noise exposure conditions were:

1. Two nights of block-randomized noise sample batteries, to assess the acute and sound pressure level dose-response effects of noise on EEG sleep (the primary study aims).
  - 20-second WFN and RTN samples, commencing only when in established sleep (N2, N3, REM), with 20 seconds of no noise between samples.
  - 3-minute WFN and RTN samples, commencing only when in established sleep, with 20 seconds of no noise between samples.
2. Three nights of WFN at typical indoor levels (WFN with amplitude modulation and infrasound at 25 dB(A)), to assess the impact of psychological influences of audible WFN on sleep time.
  - With WFN played only during wake or light sleep (N1, W).
  - With WFN played only during established sleep.
  - With WFN played throughout the sleep opportunity (from lights out until wake-up time).
3. Quiet control night (~19 dBA), as a comparator to other noise exposure conditions.

WFN samples were selected from previous recordings obtained 3.3 km from the nearest wind turbine, and included full-spectrum WFN samples including amplitude modulation with an AM depth representative of median values from year-long wind farm noise data collected at a residence 3.5 km from a wind farm. The RTN noise sample was selected from recordings obtained 700 m from a busy urban road. To help evaluate WFN infrasound effects alone, the main full spectrum WFN sample was subjected to a low-pass filter at a cut-off frequency of 15 Hz to ensure that no noise characteristics above 20 Hz would remain audible to participants with normal hearing according to ISO 226:2003 [36].

**Figure 3.**
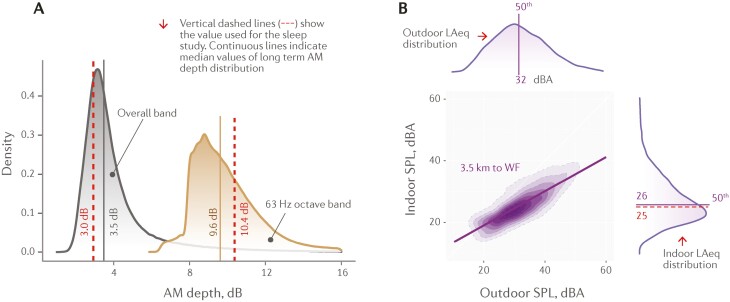
(A) Amplitude modulation depth in decibels (dB) as a function of density presented at the overall band and the 63 Hz octave band. Solid lines illustrate median values of wind farm noise data collected at a residence 3.5 km from a wind farm and perforated lines indicate the noise levels used as noise intervention stimuli in the present study. (B) LAeq distribution of outdoor versus indoor sound pressure levels (SPL) measured in A-weighted decibels (dBA) at the same residence as (A).

WFN and RTN were reproduced from existing recordings to approximate realistic noise exposure levels. Figure 3 illustrates how the AM content and SPL of this trial’s selected noise samples compare to one year long measurements at a residence in the vicinity of the same wind farm. The year-long measurements were taken at 3.5 km from the nearest wind turbine, and the trial noise samples were measured at 3.3 km from the nearest wind turbine.

Faithful reproduction of pre-recorded noise samples used an RME Babyface Pro sound card to drive a Crown DC-300 amplifier and Krix KX-4010s loudspeaker (dimensions 950 (H) × 195(W) × 295(D)mm, with a power handling range of 50–200 W) with closed vent to reproduce WFN low-frequency and infrasonic components, and a LabGruppen C 10:4X amplifier and Krix Pheonix V2.1 loudspeaker (35 Hz to 40 kHz frequency response) to reproduce traffic noise. Both speakers were placed approx. 1m away from the foot of the bed, facing the participant and ~3 m from their head. Faithful reproduction was achieved by performing 1/3-octave band equalization of the reproduced noise with the original noise samples. Noise reproduction and synchronization of acoustic and sleep recordings was controlled by custom software implemented in MATLAB (Version 2018a/b 9.4/9.5, Mathworks, Natick, Massachusetts, USA).

#### Acoustic measurements

A PROSIG P8004 24-bit Data Acquisition System and a GRAS 40AZ microphone recorded noise levels and acoustic data in each participant bedroom. The microphone was positioned ~1 meter above the participant’s head and could record noise levels as low as 17 dBA (dynamic range: 17–132 dB) and from 0.5 Hz to 20 kHz (frequency range ± 2dB).

#### Trigger cable/synchronization pulses

At noise onset and offset, a MATLAB application square pulse (200 ms long) was sent to a separate channel on the RME Babyface Pro sound card re-directed to a dedicated DC timing channel input of the polysomnography (PSG) data acquisition system (Grael 4K, Compumedics Ltd., Melbourne Australia) and sampled at 1024 Hz using Profusion 4 PSG acquisition software. Trigger pulses were also sent from the MATLAB application to the PROSIG P8004 acquisition system to further assist with accurate time-matching of independent PSG and noise recordings.

##### Primary noise interventions

**Figure 4:**
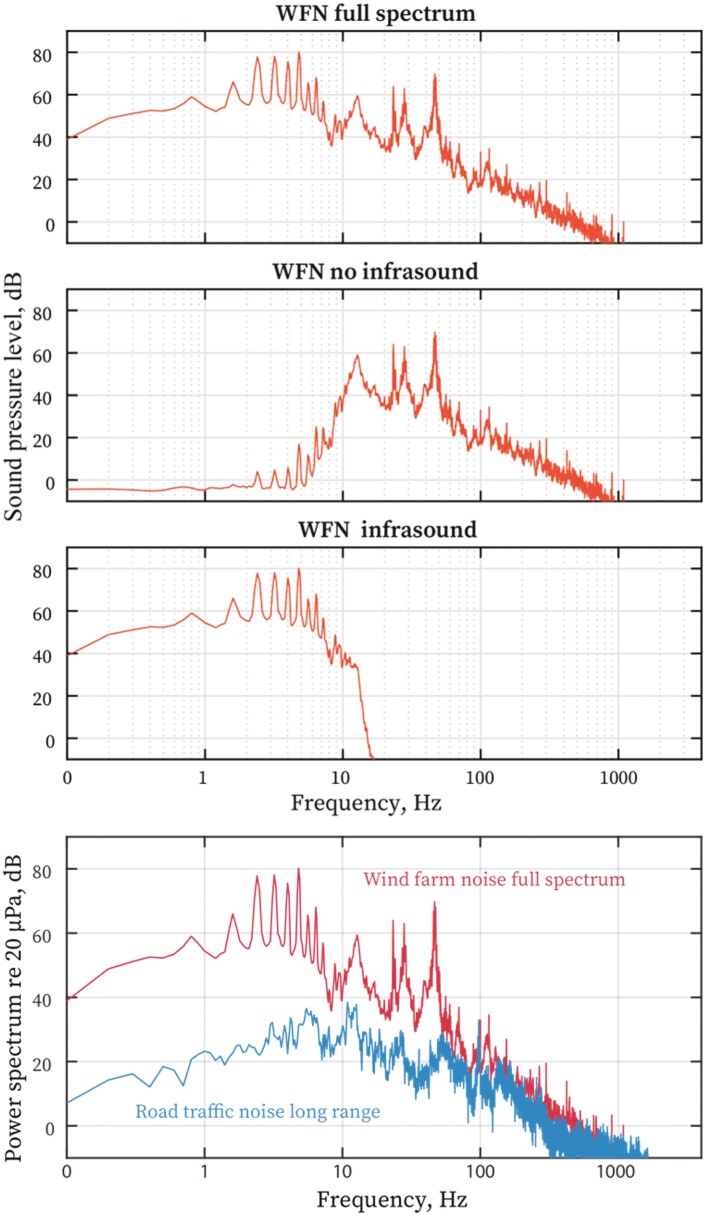
Power spectrum of wind farm and road traffic noise stimuli used as noise intervention stimuli.

The interventions to address the primary aims of the study occurred on the 20-sec and 3-min noise exposure nights. All noise interventions on these nights were played in block randomized order during N2, N3 or REM sleep. In the first instance, experimental noise exposures only commenced once 10 epochs (5 min) of PSG defined stable N2, N3 or REM sleep was initially established. In the event of an awakening or a shift to N1 sleep during any noise exposure period, the next noise sample was delayed until at least two epochs (i.e. 1 minute) of re-established N2, N3 or REM sleep. This protocol was designed to ensure that noise samples are only presented during established sleep, and to help maximize the number of repeat noise samples presented overnight. During intervention delivery, specific instructions to sleep technicians were presented on a Matlab graphical user interface to ensure technician blinding of noise types and SPLs. Specific night-to-night methods and instructions to sleep technicians are presented in Supplementary Materials (see S1). Figure 4 illustrates the power spectral content of the stimuli that were presented on the noise intervention nights. Ramp-in for these noises was 250 ms and ramp-out was < 300 ms.

###### 20-second noise battery night.

This intervention night contained samples of WFN with and without infrasound, amplitude modulation and tonality at SPLs from 30-50 dB(A), RTN at 30-50 dB(A) and background noise at 19 dB(A) in order to compare the sound pressure level dependent dose-response effects of WFN versus RTN on micro-arousal response probability (primary study aim 1). These sound pressure levels were designed to span from above to below recommended outdoor noise limits of 40 dBA at night, allowing for typical 10–15 dBA outdoor-to-indoor noise reductions with windows closed [8]. After any full awakenings, noise exposure ceased at the end of the current noise sample, until at least 1-min of sleep was re-established.

###### 3-minute noise battery night.

This intervention night contained samples of WFN (full spectra and with infrasound) at 30 and 35 dB(A), RTN at 30 and 35 dB(A) and background noise at 19 dB(A) to compare micro-arousal and awakening probability and duration with WFN versus RTN for longer noise presentations (primary study aim 2). In the event of an awakening, samples continued playing until the end of the 3-minute noise sample, after which the next sample only commenced after at least 1-min of sleep was re-established.

#### Secondary noise interventions

The secondary aim 1 to explore the effects of WFN noise exposure during wake on subsequent sleep used data from the control (quiet) night and three nights of WFN reproduced during wake and/or sleep at realistic indoor levels (at 25dB(A) with AM and infrasound), as measured inside a residence approximately 3.3km away from a wind turbine [8]. Ramp-in for the noise was 2.5 s and ramp-out was < 300 ms.

##### WFN only when awake or in light sleep.

During this condition, WFN commenced at lights out, continued whilst participants were in wake or N1 sleep, and ceased at the first signs of N2, N3 or REM sleep. Noise resumed during awakenings and continued from final awakening until lights on. The aim of this intervention night was to assess wake-dependent noise effects on EEG-derived sleep (particularly on sleep latency and wake after sleep onset) to test for potential psychological influences of audible WFN exposure during wake.

##### WFN only when asleep.

During this condition, WFN was presented following at least 10 epochs of N2, N3, or REM sleep and ceased immediately at the first signs of wake/N1 sleep, then resumed only after two epochs of re-established N2, N3, or REM sleep. This intervention night was a control night comparator to help assess for potential wake- versus sleep-dependent noise effects on EEG-evaluated sleep (particularly sleep efficiency).

##### WFN throughout the sleep opportunity.

During this condition, WFN commenced at lights out and continued until lights on. The aim of this night was to assess the overall cumulative effects of WFN on EEG-evaluated sleep when participants were continuously exposed to WFN during wake and sleep.

##### Quiet control night.

No noise samples were presented on one night to compare sleep outcomes during quiet background sleeping conditions in the same laboratory environment as the other intervention nights.

### Randomization and allocation concealment

#### Intervention night randomization

Intervention nightly conditions were randomized on Nights 2-3 (20-second noise samples and 3-minute noise samples) and Nights 4-7 (control night, WFN only when asleep, WFN only when awake, WFN throughout the sleep opportunity) separately. Individuals in both bedrooms received the same conditions. This approach was selected to help ensure primary data were collected before potential participant withdrawal from the more extended secondary study protocol and to minimize the potential for noise crossover effects between adjacent bedrooms in the sleep laboratory during noise battery nights (particularly during the quiet control and full exposure night). However, preliminary experiments confirmed that noise cross-talk between bedrooms was minimal and in the order of 2 dBA with 50 dBA noise samples. Noise recordings in both rooms were obtained to confirm the actual exposure SPLs and to assess potential extraneous noise effects.

#### Within noise battery night randomization

Noise samples presented on the 20-second and 3-minute noise sample nights were block-randomized via a “randperm(k)” function in Matlab, with k equal to the total number of samples (8 samples on the 20-second night and 9 samples on the 3-minute night).

#### Allocation concealment and blinding

To minimize potential expectation and other bias effects, the participants, experimenters in direct contact with participants, and the sleep technician manually scoring the EEG data were blinded to intervention nights and noise sample type and order. However, select study coordinators directly involved in participant screening and recruitment remained unblinded to facilitate study scheduling and adherence to the study protocol. Study participants were aware that the study was designed to compare wind farm and road traffic noise effects and were instructed that they may or may not hear a range of different noises on any given night, but remained unaware of the within and between night noise exposure design. Overnight sleep technicians responsible for delivering the noise samples remained blind to the within noise battery night randomization (i.e. they were unaware of the noise type and sound pressure level being presented to participants), but not the intervention night allocation needed to ensure that the correct intervention was administered according to the study protocol. Data were coded to permit blinding to group allocation in the primary analysis.

### Study outcomes

A complete list and detailed descriptions of all study materials are presented in the Supplementary Materials (see S2). For brevity, only the primary and secondary outcomes are described here.

#### Primary outcomes

The outcome for both primary aims to assess the dose–response effects of WFN and RTN relative to quiet background noise (control) was the presence/absence of EEG arousals (≥ 3 seconds). These were defined as the proportion of conventionally defined EEG arousal events occurring during the 20-second or 3-minute noise sample exposures.

### Study procedure

Following study consent, participants undertook two weeks of monitoring sleep at home via a daily sleep diary and actigraphy device (Philips Actiwatch 2) to establish habitual sleep timing prior to the laboratory study. Participants were asked to arrive at the sleep laboratory at 16:00 on the first night of their laboratory stay. Upon arrival, participants were inducted and familiarized with the laboratory environment, had a meal, and were set-up for a full PSG study by a trained sleep technician. Anthropometric measures were collected and a general health assessment administered, along with hair follicle collection for cortisol measurements. Once set up for PSG recording, participants completed one of eight pre-randomized listening tests and a symptoms checklist. The listening tests were designed to compare perceptual responses and measures of physiological disturbances (e.g. heart rate and vasoconstriction responses) to the range of pre-recorded WFN and RTN samples, replayed in random order. EEG was monitored throughout listening tests and if participants fell asleep, they were immediately woken up. Approximately 45-minutes prior to pre-determined lights out time, participants completed the Karolinska Sleepiness Scale [37] and the Karolinska Drowsiness Test [38].

Lights out time was set as the average bedtime reported on the previous two weeks of sleep diaries. The first laboratory night served as an adaptation night to help control for potential first-night effects of sleep study procedures in an unfamiliar environment. On successive nights, the noise interventions were administered according to the pre-randomized order. Sleep technicians monitored sleep in real-time via PSG, controlled the replay versus pause of acoustic interventions, and monitored the trigger channel to help ensure noise sample delivery according to the study protocol.

Given some variability in study setup and procedure times between nights, wake-up time was self-determined by the participant and communicated to overnight study staff prior to lights out each night. Immediately after the pre-determined wake-up time, the lights were turned on. Lights-out and wake-up times were kept as consistent as practically possible within each participant across nights. Participants were then asked to provide an initial saliva sample for cortisol measurements, and to complete a sleep diary that asked specific questions regarding their perceived sleep quality and quantity, and the noises they may have heard during the night. Four further saliva samples were taken including three taken at 15-minute intervals after initial awakening and one 12-hours after waking. After completing the sleep diary, participants completed a battery of neurocognitive tasks in the same order each morning. These comprised of a psychomotor vigilance test (PVT) [39], Karolinska Drowsiness Test [38, 40], and a Digit Symbol Substitution Test (DSST) [41]. Other psychological measures included the Karolinska Sleepiness Scale (KSS) [37] and Profile of Mood States (POMS) [42]. After testing, all monitoring devices were removed, and participants were asked to return to the laboratory by 17:30 the same day for the next evening protocol. PSG electrodes were re-applied and the same procedures were repeated across all remaining nights. During the day, participants were asked to abstain from napping and alcohol consumption, but were free to come and go from the laboratory between laboratory tests. Participants were asked to maintain their habitual levels of caffeine consumption.

## Statistical considerations

### Sample size considerations

To test the primary hypotheses, the primary analyses upon which sample size was calculated were mixed model logistic regressions with noise condition by sound pressure level interactions and binary outcomes (EEG arousal > 3 seconds). We ran a power analysis simulation for the binary outcomes defined as arousals (3-minute and 20-second nights separately) based on pilot study data [35, 43, 44], a minimum of 50 observations per participant, a two-tailed test, and 1000 simulations assuming small, medium and large effect sizes for the covariate (coefficient equal to 0.52, 1.24, and 1.90, respectively) [45] and the interaction term (beta coefficient ranging from ± 0.50 to ± 0.70 to allow for the sign of the interaction to differ from the sign of the coefficient of the exposure). The alpha was also adjusted for multiple comparisons using Bonferroni (alpha = 0.05/3). Simulations based on pilot study data from N2 sleep during the 3-minute noise battery night indicated that with a large effect size for the interaction (beta coefficient = -0.70), a sample size of 17 participants per group would provide approximately 100% power for the exposure (beta coefficient for the exposure of 2.84 for WFN versus RTN), 100% power for the covariate, and 78.8% power for the interaction term. Simulations based on the pilot study data from N2 sleep during the 20-second noise battery night indicated that 17 participants per group would provide around 100% power for the exposure (a beta coefficient of -3.24 for WTN versus RTN), 100% power for the covariate with large effect size, and 17.9% power for the interaction term with beta coefficient = 0.70. The power calculations were conducted using the ipdpower command in Stata version 16.

### Statistical analyses

All primary and secondary analyses will be two-sided with alpha set at 0.05, unless otherwise indicated. Given two primary aims tested on separate nights, the alpha level will not be adjusted for multiple primary outcomes. Secondary analyses will be exploratory, and interpreted with caution, due to multiplicity of tests and absence of pre-specified power calculations. Adjustment for multiple comparisons will be made where appropriate. No interim analyses were conducted. Effect sizes will be reported with 95% confidence intervals (95% CI). Further details are provided in the Statistical Analysis Plan in the Supplementary Materials (see S3), which details the primary analyses planned prior to analyses.

## Discussion

There is a clear need for well-designed studies using objective physiological measures to evaluate the sleep disruption characteristics of wind farm noise compared to more ubiquitous noise disturbances in sleep.

Low frequency noise effects on sleep and relationships with wakefulness perceptions of noise annoyance and acceptability have not previously been investigated in any detail, so prior data to guide study design and detailed methodological choices for this study were very limited. Hence, prior to the main laboratory trial, a pilot study was considered necessary to test noise delivery, sleep and acoustic measurement strategies, and to develop and refine signal processing methods needed to systematically evaluate noise exposure effects on sleep. Similar to the anticipated main trial, this pilot study used a within-subject, randomized, double-blind, cross-over design, consisting of 2 nights (with one week of recovery between each night) of multiple within-night noise exposures in 25 wind farm noise naïve self-reported good sleepers [35, 43, 44]. The primary aim of the pilot study was to test and refine the methods required to support this larger-scale study.

A major strength of this study is the use of current gold-standard objective sleep measures [46] in combination with a range of potentially more sensitive physiological response outcomes much less likely to exhibit bias associated with prior exposure and expectation effects compared to subjective sleep outcomes [35, 43, 44, 47–49]. Given strongly divided opinions and speculation regarding WFN, including infrasound, for which potentially placebo and nocebo effects are of concern, careful consideration of bias effects and the use of objective outcomes and study blinding to the extent that is possible are clearly important. This study will be one of the largest laboratory trials in the field of environmental noise and sleep to date. The primary outcomes are designed to help test for potential differential WFN compared to RTN effects on sleep towards better informed noise guidelines and to guide the future need and design of strategies potentially needed to help mitigate noise impacts on sleep and potentially down-stream health outcomes.

## Supplementary Material

zpad033_suppl_Supplementary_Material

## Notes

Project Location: Adelaide Institute for Sleep Health, Mark Oliphant Building, Flinders University

## Trial Registration

Australian and New Zealand Clinical Trials Registry (ANZCTR) Number: ACTRN12619000501145

Universal Trial Number: U1111-1229-6126
