# Supplementary material for: Establishing the acute physiological and sleep disruption characteristics of wind farm versus road traffic noise disturbances in sleep: a randomized controlled trial protocol
Source: Sleep Adv. 2023 Sep 6;4(1):zpad033. doi: 10.1093/sleepadvances/zpad033 (PMC10517905; doi:10.1093/sleepadvances/zpad033)
Supplement: zpad033_suppl_Supplementary_Material [file zpad033_suppl_supplementary_material.pdf]

SUPPLEMENTARY MATERIALS

Establishing the physiological and sleep disruption characteristics of wind farm versus road traffic noise disturbances in sleep: A randomised controlled trial protocol

Short title: The effects of noise disruption on sleep.

Gorica Micic, Branko Zajamsek, Bastien Lechat, Kristy Hansen, Hannah Scott, Barbara Toson,

Tessa Liebich, Claire Dunbar, Duc Phuc Nguyen, Felix Decup, Andrew Vakulin, Nicole Lovato, Leon Lack,

Colin Hansen, Dorothy Bruck, Ching Li Chai-Coetzer, Jeremy Mercer, Con Doolan, Peter Catcheside

Correspondence:
Peter Catcheside
Flinders University, Mark Oliphant Building, Adelaide Institute for Sleep Health
5 Laffer Drive, Bedford Park, South Australia 5042
Email: peter.catcheside@flinders.edu.au
Phone: +61 8 7221 8305

CONTENTS

S1: Overnight sleep technician blinding: Instructions by study night..3
S2: All Study Assessments and Timing of Administration. 10
S3: Statistical Analysis Plan. 13

S1: Overnight sleep technician blinding: Instructions by study night

ADAPTATION NIGHT

Not randomised. This night will *always* be the first in the sequence of 7 laboratory nights for all participants.

OBJECTIVE: The first-night effect is a well-known phenomenon in sleep recordings, particularly when carried out in a sleep lab or outside of the participants' usual sleep environment. The objective is to enable participant habituation and adjustment to a new sleep environment. The first night effect is generally characterised by longer stage 2 and rapid eye movement (REM) latencies, lower sleep efficiency and percentage of REM sleep. Unfamiliar surroundings (e.g., the sleep laboratory) and participants' adaptation to the application of devices and other materials applied to their body are determinants of this effect.

METHODS FOR ACOUSTICIANS:

- Sleep techs will have general knowledge of the first-night-effect and the need for an adaptation night, hence will not be blinded to the condition. This information should *not* be relayed to the participants.
- Background noise night.
- PLAY/STOP noise buttons not necessary.

The following 6 nights will be randomly allocated across participants.

CONTROL NIGHT

OBJECTIVE: Baseline night as a measure of participants' typical sleep.

METHODS FOR ACOUSTICIANS:

- Background night
- PLAY/STOP noise buttons unnecessary.

20-SECOND EXPOSURE NIGHT

OBJECTIVE: Development of physiological responses to dose-responses to short, frequent periods of noise exposure.

METHODS FOR ACOUSTICIANS:

- No noise during wake, 20-sec noise whilst asleep.
- Devise PLAY/STOP buttons to administer noise.

Phase of sleep	Noise Exposure	Tech timing	Noise timing	Button Type
**Sleep onset time**	Noise	10 epochs N2/REM	Starts immediately	PLAY
**Onset of awakenings**	No noise	Stop immediately	Stops immediately	STOP
**Return to sleep**	Noise	2 epochs n2/REM	Starts immediately	PLAY
**Final awakening**	No noise	Stop immediately	Stops immediately	STOP

INSTRUCTIONS FOR SLEEP TECHNICIANS:

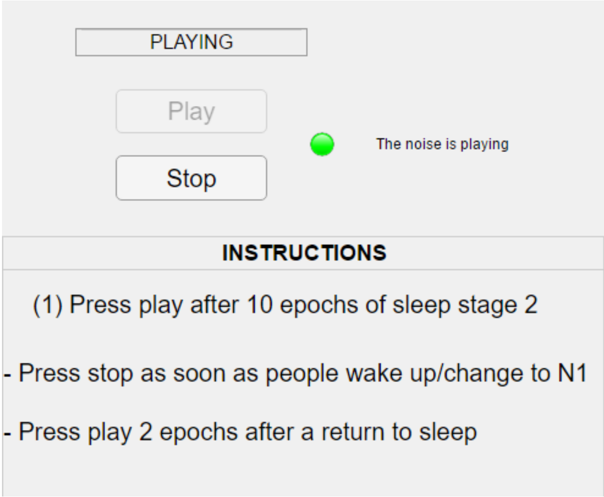

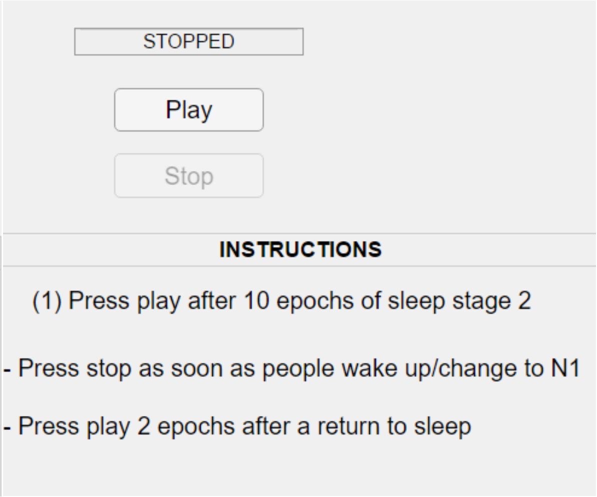

3-MINUTE EXPOSURE NIGHT

OBJECTIVE: Development of physiological responses to dose-responses to longer periods of noise exposure.

METHODS FOR ACOUSTICIANS:

- No noise during wake, 3-minute noise whilst asleep.
- Devise PLAY/STOP buttons to administer noise.

Phase of sleep	Noise Exposure	Tech timing	Noise timing	Button Type
**Sleep onset time**	Noise	10 epochs N2/REM	Starts immediately	PLAY
**Onset of awakenings**	No noise	Stop immediately	Stops 3-min after onset	STOP
**Return to sleep**	Noise	2 epochs n2/REM	Starts immediately	PLAY
**Final awakening**	No noise	Stop immediately	Stops 3-min after onset	STOP

INSTRUCTIONS FOR SLEEP TECHNICIANS:

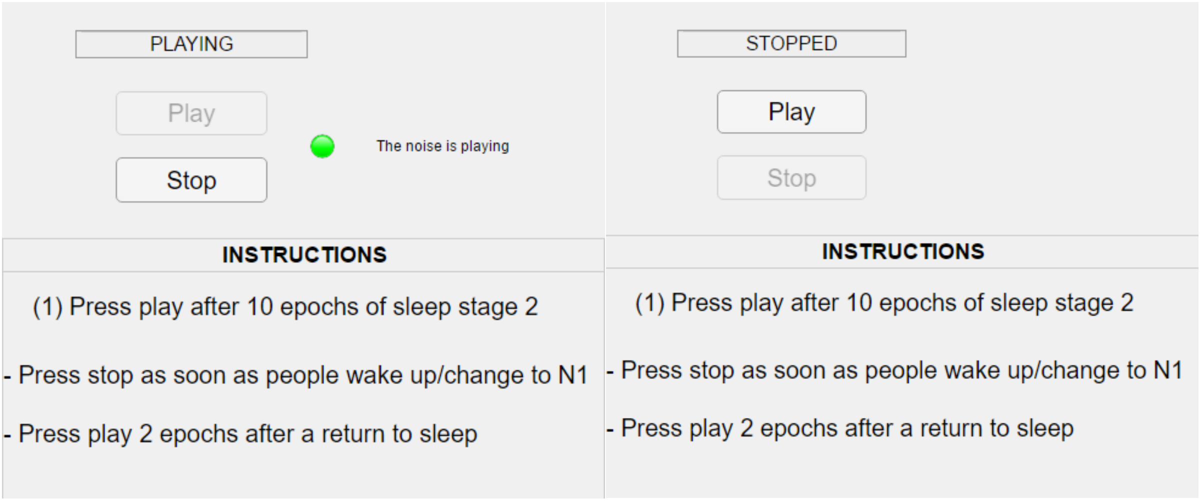

FULL NIGHT OF 25dB(A)WTN+AM

OBJECTIVE: Effects of awareness on physiological and psychological responses to noise.

METHODS FOR ACOUSTICIANS:

- Continuous night of WTN from LOT until final awakening, both when participants are asleep and wake during the night.

Phase of sleep	Noise Exposure	Tech timing	Noise timing	Button Type
**Lights out time**	Noise	10 epochs N2/REM	Starts immediately	PLAY
**Final awakening**	No noise	Stop immediately	Stops immediately	CLOSE PROGRAM

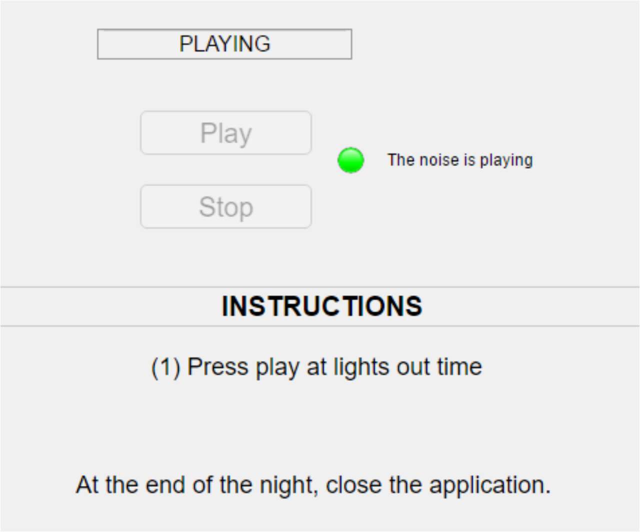

NIGHT OF 25dB(A)WTN+AM ONLY WHEN ***ASLEEP***

OBJECTIVE: Effects of awareness on physiological and psychological responses to noise.

METHODS FOR ACOUSTICIANS:

- Continuous WTN when participants are asleep and no noise when awake.

Phase of sleep	Noise Exposure	Intervention timing	Noise timing	Button Type
**Sleep onset time**	Noise	10 epochs N2/REM	Starts immediately	PLAY
**Onset of awakenings**	No noise	Stop immediately	Stops immediately	STOP
**Return to sleep**	Noise	2 epochs n2/REM	Starts immediately	PLAY
**Final awakening**	No noise	Stop immediately	Stops immediately	STOP

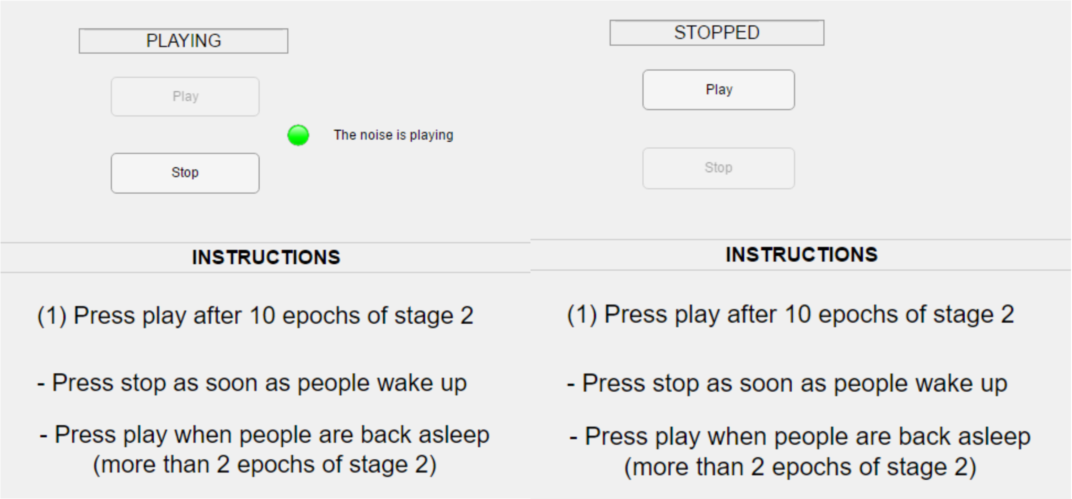

NIGHT OF 25dB(A)WTN+AM ONLY WHEN ***<u>AWAKE</u>***

OBJECTIVE: Effects of awareness on physiological and psychological responses to noise.

METHODS FOR ACOUSTICIANS:

- Continuous WTN when participants are awake and no noise when asleep.

Phase of sleep	Noise Exposure	Intervention timing	Noise timing	Button Type
**Lights out time**	Noise	Lights out	Starts immediately	PLAY
**Sleep onset time**	No noise	10 epochs N2/REM	Stops immediately	STOP
**Return to wake**	Noise	1 epoch N1/wake	Starts immediately	PLAY
**Return to sleep**	No noise	2 epochs N2/REM	Stops immediately	STOP

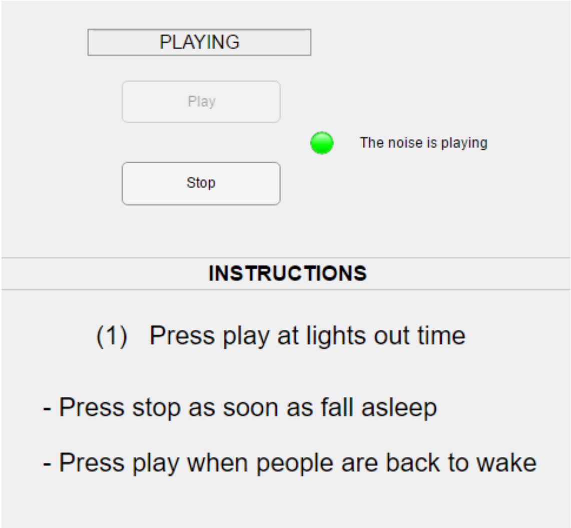

This application works the same as the night of 25db(A)WTN+AM only when asleep, only the instructions are changed.

S2: All Study Assessments and Timing of Administration.

ASSESSMENT	STUDY PERIOD						
	Screening	Pre-Laboratory Monitoring	Laboratory Protocol				Post-Laboratory Monitoring
			Adaptation Night	Mornings	Evenings	Nights 2-7	
**SCREENING ASSESSMENTS**							
General Health, Medical and Lifestyle Screening Questionnaire	**X**						
Inclusion/Exclusion Criteria	**X**						
Enrollment	**X**						
Informed Consent	**X**						
**NOISE ASSESSMENTS**							
Listening Tests			**X**		**X**		
Clinical Hearing Assessment				One daytime test			
Perceived Sleep and Noise Questionnaire				**X**			
**SLEEP MONITORING**							
Sleep diaries		**X**		**X**			**X**
Actiwatch-2 device		**X**	**X**	**X**	**X**	**X**	**X**
Polysomnography			**X**			**X**	
**BIOLOGICAL SAMPLE COLLECTION**							
Hair Cortisol Sample			**X**				
Salivary Cortisol Samples				**X**			
**HEALTH ASSESSMENTS**							
Height and weight			**X**				
Blood pressure			**X**		**X**		
Medication use			**X**		**X**		
**NEUROCOGNITIVE TASKS**							
Karolinska Drowsiness Test			**X**	**X**	**X**		
Psychomotor Vigilance Task – 10-minute				**X**			
Balance Board				**X**			
Digit Span				**X**			
Digit Symbol Substitution Task				**X**			
**QUESTIONNAIRES**							
Epworth Sleepiness Scale	**X**						
Insomnia Severity Index	**X**						
Pittsburgh Sleep Quality Index	**X**						
Functional Outcomes of Sleep Questionnaire	**X**						
Sheehan Disability Scale	**X**						
Euro Quality of Life – 5 Dimensions	**X**						

ASSESSMENT	STUDY PERIOD						
	Screening	Pre-Laboratory Monitoring	Laboratory Protocol				Post-Laboratory Monitoring
			Adaptation Night	Mornings	Evenings	Nights 2-7	
Weinstein Noise Sensitivity Questionnaire	X						
Speech Spatial Qualities – 12 items	X						
Dysfunctional Beliefs and Attitudes About Sleep Scale	X						
Ford Insomnia Response to Stress Test	X						
Depression Anxiety Stress Scale – 21 items	X						
Flinders Fatigue Scale	X						
Sleep Anticipatory Anxiety Scale	X						
Perceived Stress Scale	X						
Karolinska Sleepiness Scale			X	X	X		
Effort Scale				X			
Profile of Mood States				X			
State-Trait Anxiety Inventory			X	X			
Symptoms Checklist				X	X		

S3: Statistical Analysis Plan.

Establishing the physiological and sleep disruption characteristics of wind farm versus road traffic noise disturbances in sleep

Statistical Analysis Plan (SAP)

Version number 1.0

1. Administrative Information

1.1. Title and trial registration

Title: Establishing the physiological and sleep disruption characteristics of windfarm versus road traffic noise disturbances in sleep
Short Title: The effects of noise disruption on sleep
Coordinating Principal Investigator/ Principal Investigator: Prof. Peter Catcheside

Funding:
This research was supported by the National Health and Medical Research Council (NHMRC) Project Number: GNT1113571, 2016-2021, $1.41million
Investigators: Peter Catcheside, Leon Lack, Kristy Hansen, Con Doolan, Colin Hansen, Andrew Vakulin, Nicole Lovato, Dorothy Bruck, Jeremy Mercer, Ching Li Chai-Coetzer

Trial Registration:
Australian and New Zealand Clinical Trials Registry (ANZCTR) Number: ACTRN12619000501145
Universal Trial Number: U1111-1229-6126

Ethics:
This project has been granted ethical approval by the Southern Adelaide Clinical Human Research Ethics Committee (SAC HREC) on 03/12/2018 (Ref number 343.18)

1.2. Protocol and SAP versions

Protocol paper: Version 1.0
SAP version: Version 1.0, 31 May 2022
SAP revisions None – this SAP was finalised prior to final data cleaning and code un-blinding with no planned interim analyses.

1.3. Roles and responsibility and signatures

The undersigned have reviewed and approve this Statistical Analysis Plan.
The signatories confirm that:
1. They believe the procedures for the statistical analysis of the data described in this document are appropriate,
2. their intention is to analyse the data using the procedures described in this document and
3. if, subsequently, the statistical analyses of the data are conducted in a way that differs from the that described herein, those difference will be made explicit in reports of those analyses.

Prof. Peter Catcheside - Trial Coordinating Principal Investigator
Signature Date 1/6/2022

Dr Bastien Lechat - preparation and review of SAP document
Signature Date 01/06/2022

Dr Branko Zajamsek - preparation and review of SAP document
Signature Date 1/6/2022

Dr Gorica Micic - preparation and review of SAP document
Signature Date 6/6/22

Dr Hannah Scott - preparation and review of SAP document
Signature Date 1/6/2022

Dr Kristy Hansen - preparation and review of SAP document
Signature Date 2/6/2022

Ms Barbara Toson - preparation and review of SAP document, Statistician
Signature Date 1/06/2022

2. Introduction

2.1. Background and rationale

Sleep is essential for normal daytime functioning and health, and the effects of sleep disorders and sensory disturbances such as traffic noise on sleep quality and health outcomes are well known. Expansion of wind farm facilities in Australia has been associated with widespread community complaints regarding sleep disturbance and adverse health effects potentially attributable to wind farms operating in a normally quiet rural environment. Wind farm noise exposure, which has audible and predominantly low frequency components, including amplitude modulated noise and infrasound (<20 Hz), has the potential to adversely affect sleep, health and well-being through two main plausible and inter-related mechanisms; chronic sleep fragmentation from frequent physiological activation responses to sensory disturbances in sleep, and chronic insomnia which could potentially develop more gradually over time in sensitised individuals. However, as outlined in the NHMRC rapid review of the evidence, data from well-designed studies using objective measures of sleep and sound are remarkably lacking and are clearly now needed to definitively establish the sleep disruption characteristics of wind farm noise compared to other noise disturbances in sleep.[1]

2.2. Objectives

This study seeks to clarify the effects of wind turbine noise (WTN) on sleep compared to road traffic noise (RTN), an already known disruptor to sleep, and quiet background noise (control). The primary study aims are to compare the dose-response effects of WTN versus RTN on:

1. The probability of electroencephalographically-defined micro-arousals and awakenings from sleep (shifts to faster EEG frequencies for 3-15 seconds) under each noise condition on 20-second noise battery nights to assess the acute noise effects.
2. The probability of electroencephalographically-defined micro-arousals and awakenings from sleep under each noise condition on a 3-minute noise battery night to assess more sustained noise effects.

In addition, we will address the following secondary aims to:

1. Elucidate the role of psychological noise awareness on noise-induced objective and subjective sleep disruption, mood, anxiety, sleepiness, and daytime performance, by presenting WTN noise only during wake, only during sleep, and continuously during both wake and sleep throughout separate overnight sleep opportunities.
2. Examine the role of prior noise exposure and self-reported noise sensitivity on objective and subjective sleep in four pre-existing populations: individuals living near wind turbines with/without noise-related complaints (i.e., wind farm noise high versus low noise sensitivity), individuals living in urban areas near road traffic (active control), and individuals living in a quiet rural area.
3. Compare the dose-response effects of sound pressure level and noise type on the probability of EEG (K-complexes and quantitative electroencephalography measures) and cardiovascular activation responses (tachy-brady cardias, finger vasoconstriction and pulse arrival time).
4. Examine the dose-response effects of sound pressure level and noise type on daytime listening test outcomes of self-reported annoyance and perceived acceptability for sleep.

[1] See the Introduction of the protocol manuscript for further details on the study rationale.

2.3. Hypotheses

The hypotheses for the two primary aims are that:

1. EEG arousal responses are more probable with WTN on the 20-second noise battery night compared to RTN of equivalent A-weighted sound pressure level (SPL).
2. EEG arousals responses, including awakenings, are more probable with WTN on the 3-minute noise battery night compared to RTN of equivalent A-weighted SPL.

3. Study Methods

3.1. Trial design

This study is a randomised, cross-over, mixed-subjects (noise exposure condition: within-subjects, participant group: between-subjects), single-blind interventional trial. Four pre-existing participant groups determined by residential proximity to noise sources and reporting of noise-related complaints will be exposed to six randomised, counter-balanced overnight noise exposure conditions after an adaptation night (seven nights total).

The four participant groups are:

1) wind turbine noise exposed with noise-related complaints
2) wind turbine noise exposed without noise-related complaints
3) road traffic noise exposed with noise-related complaints, and
4) quiet rural area control.

The six noise exposure conditions are:

1) 20-second WTN and RTN noise samples only when in established sleep
2) 3-minute WTN and RTN noise samples only when in established sleep
3) WTN only when asleep
4) WTN only when awake
5) WTN throughout the sleep opportunity
6) Quiet control night.

3.2. Randomization

Intervention night randomisation

Intervention nightly conditions will be randomised on Nights 2-3 (20-second noise samples and 3-minute noise samples) and Nights 4-6 (WTN only when asleep, WTN only when awake, WTN throughout the sleep opportunity) separately. To minimise possibility of noise crossover between adjacent bedrooms in the sleep laboratory during noise battery nights (where SPL are as high as 50dB(A)), individuals participating on the same nights will be randomly allocated to the same overnight conditions.

Within Noise battery night randomisation

Noise samples presented on the 20-second and 3-minute noise sample nights will be block-randomised via a 'randperm(*k*)' function on Matlab, with *k* equal to the total number of samples (8 samples on the 20-second night and 9 samples on the 3-minute night).

Allocation concealment and blinding

To minimise potential expectation and other bias effects, the participants, experimenters in direct contact with participants, and the sleep technician manually scoring the EEG data will be blinded to intervention nights and noise sample order. However, select study investigators directly involved in

participant screening and recruitment will need to remain unblinded to ensure adherence to the study protocol. Overnight sleep technicians responsible for delivering the noise samples will be blind to the within noise battery night randomisation (i.e., they will be unaware of the noise type and sound pressure level being presented to participants), but not the intervention night allocation needed to ensure that the correct intervention is administered appropriately. Data will be coded to permit blinding to group allocation in the primary analysis.

3.3. Sample size

To test the primary hypotheses, the primary analyses upon which sample size is calculated are mixed model logistic regressions with noise condition by sound pressure level interactions and binary outcomes (EEG arousal >3 seconds). We ran a power analysis simulation for the binary outcomes defined as arousals (3 minute and 20 second nights separately) based on the pilot study data[1-3], a minimum of 50 observations per participant, a two-tailed test, and 1000 simulations assuming small, medium and large effect sizes for the covariate (coefficient equal to 0.52, 1.24 and 1.90 respectively)[4] and the interaction term (beta coefficient ranging from ±0.50 to ±0.70 to allow for the sign of the interaction to differ from the sign of the coefficient of the exposure). The alpha was also adjusted for multiple comparisons using Bonferroni (alpha=0.05/3). Simulations based on pilot study data from N2 (NREM Stage 2) sleep during the 3-minute noise battery night indicated that with a large effect size for the interaction (beta coefficient=-0.70), a sample size of 17 participants per group would provide approximately 100% power for the exposure (beta coefficient= 2.84 for WTN versus RTN), 100% power for the covariate, and 78.8% power for the interaction term. Simulations based on the pilot study data from N2 sleep during the 20-second noise battery night indicated that 17 participants per group would provide around 100% power for the exposure (an odds ratio of -3.24 for WTN versus RTN), 100% power for the covariate with large effect size, and 17.9% power for the interaction term with beta coefficient= 0.70. The power calculations were conducted using ipdpower command in Stata version 16.

3.5. Statistical interim analyses and stopping guidance

No interim analysis is planned. Noise exposure during sleep at realistic levels selected for this study are not expected to cause any adverse events, so no data safety management board or plan was constructed.

3.6. Timing of final analysis

Analyses to address the primary and secondary aims will be conducted after the date of the last signature on this document.

3.7. Timing of outcome assessments

Outcome assessments and timing windows are presented in the Supplementary Materials.

4. Statistical Principles

4.1. Confidence intervals and p values

All primary and secondary analyses will be two-sided with alpha set at 0.05, unless otherwise indicated. As the two primary aims are tested under separate nights, these are considered to be discrete experiments and thus the alpha level will not be adjusted for multiple primary outcomes. Secondary analyses are exploratory and the results will be treated with caution, due to multiplicity of tests and absence of pre-specified power calculations. Adjustment for multiple comparisons will be

made where appropriate. No interim analyses will be conducted. Effect sizes will be reported with 95% confidence intervals (95% CI).

4.2. Adherence and protocol deviations

Sleep technicians administer the noise intervention to participants from a separate room without active participant involvement, and thus, participant adherence to the intervention is not relevant to this study. Participant adherence to completing baseline measures will be assessed. Completion rate of the daily Sleep Diaries for two weeks will be reported as the percent of participants who complete at least 10/14 diary entries. The same criteria will be applied to assess adherence to wearing actigraphy devices for two weeks.

All protocol deviations will be summarised, where relevant. Definitions of protocol deviations include:

1. Noise exposures not according to protocol, due to equipment failures or other technical problems associated with noise delivery (pre-planned versus actual noise delivered).
2. Outcome measures administered outside of timing windows.

Where possible and appropriate, protocol deviations will be resolved prospectively by re-randomising noise exposure conditions or retrospectively by excluding data from the affected noise exposure conditions.

4.3. Analysis populations

All primary analyses will be conducted on an "intention-to-treat" basis. If there is substantial imbalance in dropout, crossover, or baseline variables between participants or groups, we will also perform a "per-protocol" analysis with adjustment for potential confounding variables.

5. Trial Population

5.1. Screening Data

Screening data will be evaluated for reporting as supplementary material where possible to allow for an evaluation of the population demographics from which the study sample was drawn from.

5.2. Eligibility Criteria

Inclusion Criteria

- Aged ≥18 years.
- Provides freely given informed consent.
- Able and willing to travel and remain at the laboratory for seven nights.

One of the following (to determine participant group allocation):

- Living <10km from a wind turbine and self-reporting WTN-related sleep disturbance.
- Living <10km from a wind turbine and self-reporting no WTN-related sleep disturbance.
- Self-reporting RTN-related sleep disturbance.
- Living in a quiet rural area.

Exclusion Criteria

- Language difficulties that might preclude fully informed consent.
- Self-reported sleep disorders other than Insomnia, e.g., Obstructive Sleep Apnoea, Restless Legs Syndrome.
- Pregnancy/lactation.

- Night shift work (between 10pm - 6am).
- Trans-meridian travel (equal to or greater than two time zones) in the last 2 months.

5.3. Recruitment

Eligible individuals will be recruited via advertisements posted on websites, media outlets, and public noticeboards, via community engagement talks and word of mouth, and from a sample of participants who undertook a computer assisted telephone interview (CATI) survey as part of a related project.

5.4. Withdrawal/follow-up

Attrition rate will be reported in all manuscripts. Any randomised participant who was consented to the laboratory protocol and then subsequently chose to withdraw from the study will be summarised regarding timing and their reasons for withdrawal (where available). Unless specifically requested by a participant, any data collected prior to withdrawal or lost to follow-up will be available for analysis.

5.5. Baseline patient characteristics

Baseline characteristics for the consented participants will be summarized for each participant group and include the following variables: age, sex, ethnicity, body mass index (BMI), Sleep Diary outcomes, and scores on sleep, noise sensitivity, and daytime functioning self-report questionnaires.

6. Analysis

6.1. Outcome definitions

The primary outcome for both primary aims to assess the dose-response effects of wind turbine noise (WTN) and road traffic noise (RTN) relative to quiet background noise (control) is the presence/absence of EEG arousals (>3 seconds). These are defined as the proportion/percent of this standard EEG event occurring at any time during 20-second or 3-minute noise sample exposures.

6.2. Analysis methods

For the primary aims, the interaction between noise condition and sound pressure level on the occurrence of EEG arousals will be analysed using mixed model logistic regression. All analyses will be unadjusted in the first instance and then adjusted for baseline values and participant demographics. Secondary analyses to address the secondary aims will be conducted as described above for the primary outcome analysis. Variable distributions will be examined to investigate homoscedasticity, outliers and normality, and corrected using data transformations or other suitable methods. Continuous variables which are normally distributed will be presented as means and standard deviations (SD) or confidence intervals (CI), or as medians and inter-quartile ranges for skewed data. Categorical variables will be presented as frequencies and percentages.

6.3. Missing data

Where data are missing, the number of observations will be reported. There will be no imputation of missing data for the primary outcomes. Multiple imputation of missing data will be conducted for secondary and additional analyses.

6.4. Additional analyses

The daytime listening tests examining the noise components of WTN that contribute the most to annoyance and sleep will be analysed as per the above specifications. In addition, the dose-response

effects of sound pressure level and noise type via daytime listening tests will be assessed on quantitative EEG and EEG marker outcomes.

6.5. Harms

While no harms are anticipated, incidences of all adverse events (serious adverse events, adverse events and unrelated events) will be summarised and reported.

6.6. Statistical software

Statistical analyses will be conducted in R (v 4), Stata (v 16) or IBM SPSS (v 27).

7. References

1. Lechat B, Hansen K, Micic G, et al. K-complexes are a sensitive marker of noise-related sensory processing during sleep: A pilot study. *Sleep*. Mar 12 2021;doi:10.1093/sleep/zsab065
2. Lechat B, Scott H, Decup F, et al. Environmental noise-induced cardiovascular responses during sleep. *Sleep*. 2022;45(3)
3. Dunbar C, Catcheside P, Lechat B, et al. EEG power spectral responses to wind farm compared with road traffic noise during sleep: a laboratory study. *Journal of Sleep Research*. 2022;31(3):e13517.
4. Chen H, Cohen P, Chen S. How big is a big odds ratio? Interpreting the magnitudes of odds ratios in epidemiological studies. *Communications in Statistics—simulation and Computation®*. 2010;39(4):860-864.

Correction: Establishing the physiological and sleep disruption characteristics of wind farm versus road traffic noise disturbances in sleep

In "Establishing the physiological and sleep disruption characteristics of wind farm versus road traffic noise disturbances in sleep" [Statistical Analysis Plan (SAP), Version number 1.0] the authors noted one error in section 3.3 Sample size.

The word odds ratio was used in place of log odds ratio (beta coefficient).

"Simulations based on the pilot study data from N2 sleep during the 20-second noise battery night indicated that 17 participants per group would provide around 100% power for the exposure (an odds ratio of -3.24 for WTN versus RTN) …"

Should instead read as

"Simulations based on the pilot study data from N2 sleep during the 20-second noise battery night indicated that 17 participants per group would provide around 100% power for the exposure (a beta coefficient of -3.24 for WTN versus RTN) …"

The change does not affect the power calculation which were done using the beta coefficient value.

Because this error was noted after finalising the SAP and does not alter the content, the current correction notice is signed and dated separately, and saved in the same folder. The corrected terminology will be used in the protocol paper and any other subsequent article.

Prof. Peter Catcheside - Trial Coordinating Principal Investigator
Signature Date 28/07/2022

Dr Bastien Lechat - preparation and review of SAP document
Signature Bastien Lechat Date 28/07/2022

Dr Branko Zajamsek - preparation and review of SAP document
Signature Date 28/07/2022

Dr Gorica Micic - preparation and review of SAP document
Signature Gorica Micic Date 01/08/2022

Dr Hannah Scott - preparation and review of SAP document
Signature Hannah Scott Date

Dr Kristy Hansen - preparation and review of SAP document
Signature Kristy L Hansen Date 28/07/2022

Ms Barbara Toson - preparation and review of SAP document, Statistician
Signature Barbara Toson Date 28 July 2022
